# Influence of Supplemental Feed Choice for Pasture-Based Cows on the Fatty Acid and Volatile Profile of Milk

**DOI:** 10.3390/foods8040137

**Published:** 2019-04-22

**Authors:** Tom F. O’Callaghan, David Mannion, Diana Apopei, Noel A. McCarthy, Sean A. Hogan, Kieran N. Kilcawley, Michael Egan

**Affiliations:** 1 Food Chemistry and Technology, Teagasc Moorepark Food Research Center, Moorepark Fermoy, P61 C996 Co. Cork, Ireland; david.mannion@teagasc.ie (D.M.); diana.apopei@teagasc.ie (D.A.); Noel.mccarthy@teagasc.ie (N.A.M.); Sean.A.Hogan@teagasc.ie (S.A.H.); Kieran.kilcawley@teagasc.ie (K.N.K.); 2 Teagasc Animal and Grassland Research, Moorepark Fermoy, P61 C996 Co. Cork, Ireland; Michael.Egan@teagasc.ie

**Keywords:** dairy, fatty acids, pasture, feed supplements, milk composition, cow diet

## Abstract

The purpose of this study was to examine the impact of a variety of supplemental feeds on the composition and quality of milk in a pasture-based dairy system. Four pasture-supplemented feeding systems were compared: Group 1 supplementation with 16% crude protein parlour concentrate (CONC); Group 2 supplementation with palm kernel expeller plus parlour concentrate (PKE); Group 3 supplemented with soya hulls plus parlour concentrate (SOYA); Group 4 was supplemented with molassed beet pulp plus parlour concentrate (BEET). Supplemental feeding system was demonstrated to have a significant effect on the size of native casein micelles and the gelation properties of milks. While CONC feeding produced significantly higher casein micelle size, gel strength (Young’s Modulus) was significantly negatively correlated with casein micelle size. Supplemental feeding system had a significant effect on a number of fatty acids (FA) and indices derived therefrom, including total saturated and unsaturated fatty acids, *de novo* produced FA, omega 3, and omega 6 FA. The volatile profile of milks was also affected by supplemental feed choice, whereby multivariate analysis demonstrated that the CONC diet was distinctly different to that of the PALM, SOYA, and BEET milks. Multivariate analysis demonstrated that it is possible to distinguish milks from different pasture-supplemented feeding systems by their FA profile.

## 1. Introduction

Milk and dairy products are highly nutritious food stuffs for individuals throughout all stages of life. The composition of milk directly influences the quality, yield, functionality, and nutritional value of milk and dairy products. A number of factors affect the composition of milk namely, breed, stage of lactation, health status of the cow, and diet. Dietary factors, in particular, affect the composition and nutritional content of milks through manipulation of the fatty acids (FA) profile, while cow breed can influence the composition of milk proteins. Therefore, selection of appropriate farm management practices offers one way of modulating milk composition and processing properties. Extensive research has been carried out examining the effects of pasture or conventional total mixed ration (TMR) on the composition and quality of milk and dairy products. Pasture-based feeding of cows, which is estimated to account for ~10% of the global milk supply [1], has been demonstrated to have beneficial effects on the nutritional profile of milk. These include significant decreases in the proportion of saturated fatty acids (SFA), particularly C16:0, and omega 6 FAs, and increased amounts of polyunsaturated fatty acids (PUFA), along with nutritionally beneficial FAs such as conjugated linoleic acid, branched chain, and omega 3 FA [2,3]. Indeed, Couvreur et al. [4] demonstrated a linear response for many of these nutrients with increasing proportion of pasture in the cow diet. Pasture-feeding induced alterations to the FA profile of milk and also had a significant effect on the volatile and sensory properties of milk [5], and composition and quality of products such as sweet cream butter [6] and full-fat cheddar cheese [7]. White et al. [8] demonstrated a significant effect between warm season pasture feed versus TMR feed on milk fatty acid profile, but also highlighted that cow breed can have an influence on the fatty acid profile of milks.

Several factors determine the suitability of dairy feeding systems to particular regions of the world namely, climate, environmental conditions, soil properties, availability of grazing lands, and dietary requirements of the animals. Ireland has a predominantly temperate climate with plentiful rainfall providing ideal conditions for the growth of pastures and with that a seasonal pasture-based dairy system. As such, O‘Brien et al. [9] demonstrated that forage, particularly grazed pasture, was the largest component (76%) of the Irish cows annual diet as measured over a three-year period. There are a wide variety of benefits associated with the use of pasture-based feeding systems, including improved consumer perceptions of pasture derived products as being a healthy and animal friendly, improved environmental sustainability, and a cheap source of highly nutritious feed for the cow compared to conventional TMR practises. There are, however, also certain limitations to the use of pasture-based feeding systems, which can include, but are not limited to, a seasonal rather than annual supply of milk to producers, “peak” milk production periods, seasonal based changes in milk composition, and limitations of grass growth during certain periods of the year. Given this dependence of pasture availability on climate and environment, there are periods of the year, and potential for non-typical weather occurrences such as drought, when pasture-based systems will require input of concentrate supplements to meet the energy requirements of high yielding cows. Choice of supplemental feed source will inevitably affect the composition, quality, and processability of milk. Therefore, greater knowledge is required on the impact of the different supplement choices on milk quality and usability, to allow farmers and manufacturers to make more informed decisions taking into account subsequent downstream effects on product quality. There is currently limited information available comparing the effects of a variety of supplemental feeding choices to a pasture-based system on the composition and quality of milks. While there is a wide range of supplemental feed types readily available, data trends have indicated an increase in the import of soya hulls, palm kernel expeller, and beet pulp to Ireland between 2012 and 2018, indicative of their increased levels as choice of supplement. The purpose of this study was to examine the effects of supplementing a perennial ryegrass pasture-fed dairy system in Ireland with parlour concentrate, and three by-products; palm kernal, soya hulls, or molassed beet pulp on the macro composition, fatty acid profile, and some functional properties of milk.

## 2. Materials and Methods

### 2.1. Reagents

Hexane, heptane, and 25% sodium methoxide were purchased from Sigma Aldrich (Dublin, Ireland). Diethyl ether was purchased from Fisher Scientific (Dublin, Ireland). Internal standard trinonadecanoin (C_19:0_) (part number: T-165) and a standard mix of conjugated linoleic acid C_18:2,c9t11_ and C_18:2,c10t12_ (part number: UC-59M) were purchased from Nu-Chek-prep, Inc. (Elysian, MN, USA). Fatty acid methyl ester (FAME) standard mix containing C_4:0_–C_24:0_ methyl esters (part no: 35077) were purchased from Thames Restek UK Ltd (Buckinghamshire, UK). Sodium hydrogen monohydrate and glucono delta-lactone (GDL) were purchased from Sigma Aldrich (Dublin, Ireland). 

### 2.2. Experimental Design

Sixty spring-calving Friesian and crossbred (Jersey x Friesian) cows were allocated to one of 4 groups (*n* = 15) at the Teagasc Animal and Grassland Research and Innovation Centre, Moorepark, Fermoy, Co. Cork, Ireland. The experiment was conducted over a seven-week period (23 July to 31 August 2018). Cows were selected and blocked of mean (±SE) for; calving date 6 February 2018 (±1.14), parity 2.2 (±0.04), milk yield 21.2 (±0.40) kg/day, milk fat 43.3 (±1.70) g/kg, milk protein 34.9 (±0.60) g/kg, daily milk solids 1.65 (±0.04) kg/day, and 159 (±21.4) days in milk. Four feeding systems were compared over the seven-week period (Table 1). Group 1 was fed 2 kg/cow per day of a 16% crude protein parlour concentrate (CONC), Group 2 was fed 4 kg/cow per day palm kernel expeller plus 2 kg/cow per day parlour concentrate (PALM), Group 3 was fed 4 kg/cow per day soya hulls plus 2 kg/cow per day parlour concentrate (SOYA), Group 4 was fed 4 kg/cow per day molassed beet pulp plus 2 kg/cow per day parlour concentrate (BEET). The parlour concentrate diet consisted of soybean meal (300 g/kg fresh weight), beet pulp/molasses (155 g/kg), barley (150 g/kg), maize (130 g/kg), maize distillers (120 g/kg), rapeseed meal (75 g/kg), Megalac (33 g/kg), maize/beet (25 g/kg), acid buff (7 g/kg), and salt (5 g/kg); the crude protein (CP) content was 160 g/kg fresh weight. The cows with additional supplementation (Groups 2, 3, and 4) were fed after morning milking (08:30) in individual feeders. Cows consumed ~5 kg of dry matter per day (DM/d) (see Table 2 and Table 3) of grazed grass, measured by pre- and post-grazing sward heights daily using the rising plate meter (Jenquip, Feilding, New Zealand), whereas pre-grazing herbage mass was measured with an Etesia mower (Etesia UK Ltd., Warwick, UK). Cows in Group 1 consumed ~9 kg of DM/d and cows in groups 2, 3, and 4 consumed ~5 kg of DM/d of grass silage measured by the difference between offered and refusal weights. Cows on the farm were milked at 07:30 and 15:30 daily, and milk yields were recorded using DairyMaster milk meters (DairyMaster, Kerry, Ireland). In week 6 and 7 of the experimental period, bulk milk samples were collected from the morning milking only from each of the feeding systems. To achieve this, cows in each of the 4 feeding systems were segregated in the milking parlour and were milked separately into designated refrigerated tanks. Bulk milk samples only were collected post morning milking on each of the sampling days (*n* = 3) and stored at 4 °C before analysis; a subset of samples were also frozen at −20 °C prior to analysis.

### 2.3. Milk and Feed Compositional Analysis

Total nitrogen (TN), crude protein (CrP), non-protein nitrogen (NPN), non-casein nitrogen (NCN), and true protein (TP) were determined as outlined by the International organisation for standardization ISO [10,11] using the Kjeldahl method and a nitrogen-to-milk protein conversion factor of 6.38. These N values were then used to calculate true protein (TP), casein protein (C_p_), and whey protein (W_p_) contents as outlined by Auldist et al. [12] where TP = TN-NPN × 6.38, C_p_ = (TN-NCN) × 6.38 and W_p_ = (NCN-NPN) × 6.38. Milk samples were analysed for fat, lactose, and total solids contents by infrared absorption spectroscopy using a FT6000 Milkoscan (Foss Ireland Ltd, Dublin, Ireland). Milk yield from each of the diets are shown in Table 4. Feed samples were collected throughout lactation from paddocks at time of grazing. Samples were dried at 60 °C for 48 h, milled, and stored prior to analysis. Grazed grass and offered silage samples were analysed using near infrared reflectance spectroscopy using a FOSS 6500 (FOSS Ireland Ltd, Dublin, Ireland). Feed supplement samples during the trial were bulked to one sample per diet and analysed for the major FAME by Dairygold Analytical Services Laboratory (Lombardstown, Mallow, Co. Cork, Ireland) (Table 5).

### 2.4. Milk Fatty Acid Analysis

Lipid Extraction. Lipid extraction was performed as per the procedure outlined by De Jong and Badings [13]. Briefly, 10 mL of milk were added to 10 mL of ethanol (98% purity), and 1 mL of 2.5 M H_2_SO_4_ was added to each sample mixture. This mixture was extracted three times with 15 mL diethyl ether/heptane (1:1) and each time the solution was clarified by centrifugation at 1500× *g* for 5 min. The collected extracts were pooled and dried down at 55 °C under N gas.

Methyl Ester Derivatisation of Triglycerides. A volume of 4.8 mL of C19:0 TAG (500 ppm) in heptane was added to 60 mg of the extracted lipid sample, after which 200 µL of 2 M sodium methoxide solution was added and the sample was mixed vigorously for about 30 s. Then, 1 g of sodium hydrogen sulfate monohydrate (Sigma Aldrich, Dublin, Ireland) was added to the solution and shaken vigorously. After the salt had settled, the upper layer containing the methyl esters was poured into a clean test tube and diluted with 8 mL of heptane. Fatty acid methyl esters (FAME) were stored at −20 °C prior to GC analysis in 2 mL amber vials which were capped with polyetrafluoroethylene (PTFE) white silicone septa.

Instrument Conditions for Analysis of FAME. Fatty acid methyl esters analysis was performed on an Agilent 7890A gas chromatograph, equipped with an Agilent 7693 autosampler (Agilent Technologies, Cork, Ireland) and flame ionisation detector. The column was a Select FAME capillary column (100 m × 250 µm Internal diameter (I.D.), 0.25 µm phase thickness, part number: CP7420) (Agilent Technologies, Little Island, Cork, Ireland). The injector was held at 250 °C for the entire run and was operated in split mode using a split ratio of 1:10. The inlet liner was a split gooseneck liner (Part no.: 8004–0164, Agilent Technologies). The column oven was held at 80 °C for 8 min and raised to 200 °C at 8.5 °C/min, and held for 55 min. The total runtime was 77.12 min. The FID was operated at 300 °C. The carrier gas was hydrogen and was held at a constant flow of 1.0 mL/min. Results were processed using OpenLab CDS Chemstation edition software version Rev.C.01.04 (35) (Agilent Technologies).

Standard curves for FAME analysis along with in-run quality control samples were prepared using an Agilent 7696A Sample Prep Workbench instrument (Agilent Technologies, Little Island, Cork, Ireland).

### 2.5. Nutritional Indices and Fatty Acid Ratios

Several FA’s ratios and nutritional indices of milks from each of the feeding systems are reported. The summation of Ω 6 and Ω 3 and Ω 9 are reported. The atherogenecity index (AI) and thrombogenecity index’s (TI) outlined by Ulbricht and Southgate [14] are dietary risk indicators for cardiovascular disease. AI is based on the ratio of fatty acids with pro-atherogenic and those with anti-atherogenic properties. It is indicative of the propensity to inhibit aggregation of plague and diminish the levels of esterified FA, cholesterol, and phospholipids, thereby preventing the development of micro and macro-coronary diseases; while TI is based on the relationship between pro-thrombogenic and anti-thrombogenic fatty acids, and is indicative of the tendency to form clots in the blood [15].

Atherogenecity index (AI) and thrombogenicity index (TI) were calculated as described by Ulbricht and Southgate [14];

(1) $$\mathrm{AI}=\frac{C12:0+\left(4\times C14:0\right)+C16:0}{n6\mathrm{PUFA}+n3\mathrm{PUFA}+\mathrm{MUFA}}$$

(2) $$\mathrm{TI}=\frac{C14:0+C16:0+C18:0}{\left(0.5\times \mathrm{MUFA}\right)+\left(0.5\times n6\mathrm{PUFA}\right)+\left(3\times n3\mathrm{PUFA}\right)+\left(\frac{n3\mathrm{PUFA}}{n6\mathrm{PUFA}}\right)}.$$

### 2.6. Milk Colour Analysis

Milk colour was analysed using Minolta Chroma Meter CR-400 m (Mason Technology Ltd, Dublin, Ireland). Raw milks were filled into cuvettes to the indicated mark and five replications of the L, a*, and b* values were taken from random locations across the surface of the cuvette. The mean of the five replications was calculated and used as a unit for one replicate of said milk sample in statistical analysis. L value defines the position of the sample on the lightness–darkness axis, a* on the green–red axis, and b* on the blue–yellow axis [16].

### 2.7. Volatile Analysis

Milk sample (3 g) was added to a 20 mL amber screw-capped solid phase microextraction (SPME) vial (Apex Scientific, Maynooth, Ireland) and equilibrated to 40 °C for 10 min with pulsed agitation of 5 s at 500 rpm. Sample introduction was accomplished using a Bruker CTC autosampler (Elementec Ltd, Maynooth, Ireland). A single 50/30 um Carboxen^TM^/divinylbenzene/polydimethylsiloxane (DVB/CAR/PDMS) fibre was used (Agilent Technologies Ltd, Cork, Ireland). The SPME fibre was exposed to the headspace above the samples for 20 min at depth of 1 cm at 40 °C. The fibre was retracted and injected into the GC inlet and desorbed for 2 min at 250 °C. Injections were made on a Scion 456-GC (Elementec Ltd, Maynooth, Ireland) with an DB-624 Ultra inert (UI) (60 m × 0.32 mm × 1.8 μm) column (Agilent Technologies Ltd, Cork, Ireland) using a split/splitless injector with a 1/10 split. A merlin microseal (Merck, Arklow, Ireland) was used as the septum. The temperature of the column oven was set at 40 °C, held for 5 min, increased at 5 °C/min to 230 °C, then increased at 15 °C/min to 260 °C, yielding at total GC run time of 65 min. The carrier gas was helium held at a constant flow of 1.2 mL/min. The detector was a Scion EVOQ triple quadrapole mass spectrometer detector (Elementec Ltd, Maynooth, Ireland), ran in single quad mode. The ion source temperature was 220 °C and the interface temperature was set at 260 °C. The MS mode was electronic ionization (70 v) with the mass range scanned between 35 and 250 amu. Compounds were identified using mass spectra comparisons to the NIST 2014 mass spectral library and an in-house library created using authentic compounds with target and qualifier ions and linear retention indices for each compound, using Bruker MS Workstation 8 software. Retention indices were calculated (according to van Den Dool and Dec. Kratz [17]) and matched against peer reviewed publications where possible, to confirm compound identification. Spectral deconvolution was also performed to confirm identification of compounds using AMDIS. An auto-tune of the Gas chromatography mass spectrometry (GCMS) was carried out prior to the analysis to ensure optimal GCMS performance. A set of external standards was run at the start and end of the sample set and abundances were compared to known amounts to ensure that both the SPME extraction and MS detection was performing within specification.

### 2.8. Measurement of Native Casein Micelle Size

Milk samples were defrosted at refrigerated temperatures 24 h prior to analysis. The samples were defatted by centrifugation at 5000× *g* for 5 min and subsequent removal of the fat layer. The measurements on native casein (CN) micelle size were performed using a ZetaSizer Nano ZS apparatus (Malvern Instruments Ltd., Malvern, UK). The diluted samples were filtered through a 0.45-μm filter (Millex-HV, Merckmillipore) before measurement and each sample was run in triplicate. Zetasizer nano parameters were set at a dispersant refractive index (RI) of 1.330 and viscosity (Cp) of 0.8872. Solutions were measured in a disposable sizing cuvette and the material was characterised as protein with an RI of 1.45. Native CN micelle sizes were determined at room temperature. Data was displayed as the z-average (d.nm), which correlates to the intensity weighted mean size of all particles present in the dispersion analysis.

### 2.9. Gelation Properties of Raw Milks

To examine the effect of diet on the gelation properties of milks, 4% glucono delta-lactone (GDL) was added to whole raw milk and stirred for 2 min at 30 °C. Small amplitude oscillation measurements were carried out using a Discovery HR1-Hybrid rheometer, equipped with concentric cylinder geometry, maintained at 30 °C. An aliquot (17 g) of the acidified milk was weighed into the concentric cylinder. A time sweep was carried out using the following procedure: 5 s temperature soak at 30 °C, 15 s pre-shear at a shear rate of 50 s^−1^ and 10 s equilibration, followed by oscillation at 0.5% strain, at a frequency of 1 Hz and a 10 s sampling interval. The remaining acidified sample was incubated in a water-bath at 30 °C, the pH of which was continuously monitored until pH 4.6 was reached. The time sweep was then stopped and followed immediately by a logarithmic frequency sweep from 1 to 100 Hz at a constant strain of 1%.

### 2.10. Statistical Analysis

Statistical analysis was carried out using SPSS v18.0 (IBM statistics Inc, Armonk, NY, USA). Datasets were analysed for normality using the Shapiro–Wilks test. Data was found to be normally distributed and was analysed using one-way ANOVA with post-hoc Tukey test. Correlation analysis was carried out using Pearson’s Correlation Coefficient analysis.

Multivariate data analysis of fatty acids and volatiles was carried out using principal component analysis (PCA) and was carried out using the FACTOEXTRA package in R. One-way ANOVA with post-hoc Tukey analysis of volatile compounds was carried out using MetaboAnalyst [18].

## 3. Results and Discussion

### 3.1. Milk Chemical Composition

No significant effect of diet was observed on the macro composition of bulk milks (Table 6). Among the N fractions, there was a significant effect of diet on the non-casein N (NCN) content of milks (*P* = 0.014) whereby BEET NCN was significantly lower than that of PALM (*P* = 0.03) and CONC (*P* = 0.020) samples. This difference observed in the NCN also resulted in similar significant differences in the calculated % whey protein contents. BEET whey content was significantly lower than that of PALM (*P* = 0.003) and CONC (*P* = 0.001) samples; the whey content of SOYA was also significantly lower than that of CONC (*P* = 0.014).

### 3.2. Milk Fatty Acid Composition

Feeding system was shown to have a significant effect on the majority of fatty acids analysed, as is shown in Table 7 and Table 8.

The fatty acid composition of milk is typically derived from two main sources, through *de novo* synthesis and via uptake of preformed fatty acids [19]. *De novo* milk fatty acids C4–C14 are synthesised by the cow’s mammary gland. The substrates for *de novo* synthesis are acetate and ß-hydroxybutyrate, which are products of rumen fiber digestion [19]. Therefore, the content of *de novo* fatty acids in milk has in recent years received a renewed interest as a result of its use as a potential indicator of rumen functioning in the herd [20]. Diet was shown to have a significant (*P* ≤ 0.001) effect on the *de novo* fatty acid content of milks. The PALM diet resulted in milk with the highest level of *de novo,* FA significantly higher than that of BEET, SOYA, and CONC (*P* ≤ 0.001). However, the increased concentration of C12:0 and C14:0 in PALM diet likely resulted in increased levels of absorption of these fatty acids being reflected in the milks.

Diet was shown to have a significant effect on the total proportion of saturated fatty acids (SFA), which was highest in PALM and lowest in the CONC milk. CONC milk had significantly lower SFA than that of BEET (*P* = 0.044) and PALM (*P* = 0.006), while SOYA also had significantly lower SFA than that of PALM (*P* = 0.019). Dairy fat has been the subject of negative consumer attention in the past particularly attributed to its high levels of SFA. However, while particular SFA such as lauric, myristic, and palmitic acid in isolation can increase low-density lipoprotein (LDL)-cholesterol concentrations, milk is a matrix of other components that have an array of potential benefits including increasing the concentration of high-density lipoprotein (HDL)-cholesterol [21,22]. In fact, meta-analyses of the topic have concluded that milk and dairy products consumption have at least a neutral effect on health outcomes [23], and may protect against prevalent chronic diseases [24]. However more recent data suggests health considerations of products based on SFA alone can often be misinformed. The level of SFA in milk has significant implications for the functional characteristics and processability of high-fat dairy products. Recent studies have demonstrated that increased SFA (particularly C16:0) results in significant alterations to hardness and textural properties of dairy products [4,6]. The C16:0 content was highest in BEET-derived milk, which was significantly greater than that of PALM milk (*P* = 0.013). Palmitic acid (C16:0) is the most abundant SFA in bovine milk; increased proportions of C16:0 in BEAT-derived milks corresponds with higher contents of C16:0 in the BEET diet (Table 5).

Among total unsaturated fatty acids (UFA), CONC diet resulted in significantly higher UFA than that of PALM (*P* = 0.001). PALM diet resulted in milk with significantly lower poly unsaturated fatty acids (PUFA) than that of BEET, CONC, and SOYA (*P* ≤ 0.001). Among dairy PUFA, the isomer conjugated linoleic acid (CLA) c9t11 has been studied for potential health benefits and biological functions, which include impacting immune function and protective effects against cancer, obesity, and atherosclerosis [25]. The fatty acid CLAc9t11 is produced via the biohydrogenation of linoleic acid in the rumen by rumen microorganisms [26]. Therefore, CLAc9t11 in milk is highly dependent on the cow feeding system used [27] and the level of fatty acid substrate available has a direct effect on the its subsequent levels in milk. PALM feeding of cows resulted in milk with significantly reduced CLAc9t11 than that of BEET (*P* = 0.002), CONC (*P* ≤ 0.001), and SOYA (*P* = 0.001) milks. This result can be attributed to the substantially lower levels of linoleic acid in the feed supplement of PALM compared to the other diets (Table 5).

Diet did not have a significant effect of the total level of mono-unsaturated fatty acids (MUFA) of milk (*P* = 0.08). Among the total content of short-chain fatty acids (SCFA), PALM milk had significantly higher SCFA than that of BEET, CONC, and SOYA (*P* ≤ 0.001). Among the medium-chain fatty acids (MCFA), BEET milk had significantly higher MCFA than that of PALM milk (*P* = 0.014). Among long-chain fatty acids (LCFA), the CONC diet produced milk with highest content of LCFA, significantly higher than that of BEET (*P* = 0.021). However, while increased levels of LCFA can have a positive effect on milk fatty acid profile, increased levels of unsaturated LCFA can have a negative effect on the oxidative stability of milk fat [28].

Given changes in nutritional perceptions of milk and milk fatty acids as a whole, it could be suggested that judging dairy products by their total SFA content alone can be misinformed and erroneous, while other indices such levels of Omega (Ω) 3 and Ω 6 fatty acids should also be considered. As discussed by Patterson et al. [29], both Ω 3 and Ω 6 are essential fatty acids and precursors to anti-inflammatory and pro-inflammatory eicosanoids respectively. However, dietary changes in line with the western diet has resulted in an increase of Ω 6 fatty acids in the diet coinciding with increases in prevalence of chronic inflammatory diseases. CONC feeding was demonstrated to produce milks with highest level of Ω 3 fatty acids. PALM feeding, however, resulted in milk with significantly lower Ω 3 content than that of CONC (*P* ≤ 0.001), BEET (*P* = 0.002), and SOYA (*P* = 0.001). PALM feeding also produced milk with significantly lower Ω 6 than that of BEET (*P* = 0.017) and SOYA (*P* = 0.005). The thrombogenic (TI) and atherogenic (AI) indices are both dietary risk indices for cardiovascular disease. CONC milk had the lowest AI of each of the diets and was significantly lower than that of BEET (*P* = 0.031) and PALM (*P* = 0.004). The SOYA diet also resulted in a significantly lower AI than that of PALM (*P* = 0.022).

**Figure 1:**
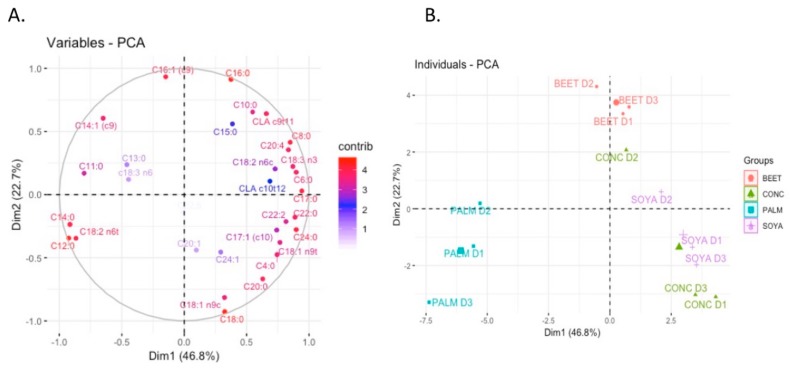
Principal component analysis of fatty acid methyl esthers (FAME) compounds detected from milks from each of the diets, parlour concentrate (CONC), palm kernel expeller (PALM), soya hulls (SOYA), and molassed beet pulp (BEET), principal component analysis (PCA).

Multivariate analysis of the fatty acids of milks has previously been shown capable of distinguishing milks from pasture versus conventional feeding systems [3]. Similar results have been shown in this study where there is clear separation between the SOYA-, BEET-, and PALM-derived milks. However, each of the diets appeared to share a similar composition with that of the CONC. This is perhaps to be expected given the similarities between the base ingredients of CONC and other diets composition (Figure 1). The top 10 fatty acids for the observed separation in Figure 1 are shown in Supplementary Figure S1, which include C16:0, C12:0, C18:0, C14:0, C16:1, C8:0, C17:0, C24:0, C18:2n6t, and CLA c9t11. Lauric acid was highest in PALM-derived milks, significantly higher than that of CONC, BEET, and SOYA (*P* ≤ 0.001). While C12:0 can be produced by *de novo* synthesis, the levels of the fatty acid in the diet can have a direct effect on its concentrations in the final milks as was demonstrated by Hristov et al. [30], who reported supplementation of C12:0 doubled its concentrations in raw milk. Increased levels of C12:0 in PALM milk corresponds with increased levels in the PALM feed compared to that of the other diets (Table 5). Stearic acid (C18:0) could be referred to have a neutral effect on human health, as it has not been shown to increase total cholesterol or LDL-cholesterol concentrations [31]. BEET-derived milks had significantly reduced concentrations than that of CONC (*P* = 0.019) and SOYA (*P* = 0.047). Myristic acid, along with palmitic acid, have been demonstrated to have cholesterol increasing properties [32]. Diet had a significant effect on the milk content of myristic acid (C14:0) where it was significantly higher in PALM than that of BEET, SOYA, and CONC (P ≤ 0.001). CONC feeding resulted in the lowest level of C14:0 in milk, which was also significantly lower than that of BEET (*P* = 0.044). Such results follow similar trend to the level of C14:0 being provided by the diet. These results are in agreement with those of Hristov et al. [30], who reported an increase in C14:0 in milk from cows supplemented with myristic acid. Caprylic acid (C8:0) was lowest in PALM milk, significantly lower than CONC (*P* = 0.002), SOYA (*P* ≤ 0.001), and BEET (*P* ≤ 0.001). Levels of odd and branched chain have been receiving increased attention. In particular, C15:0 and C17:0 have been used as biomarkers of intake of ruminant fat by humans [32]. Odd and branched-chain fatty acids such as C15:0 and C17:0 are derived from *de novo* synthesis in the mammary gland from propionate in ruminants, and diet of cows has been demonstrated to have a significant effect on their concentrations in milk [32]. C17:0 was lowest in the milks in PALM-derived milks, significantly lower than CONC, BEET, or SOYA (*P* ≤ 0.001).

O’Callaghan et al. [3] and Capuano et al. [33] found that milk fatty acid profiling could be used as a potential tool for verification of cow feeding system, and as such these results progresses this concept in that it is also capable of distinguishing small changes in the pasture-based diet such as the inclusion of supplements.

### 3.3. Milk Volatile Analysis

**Figure 2:**
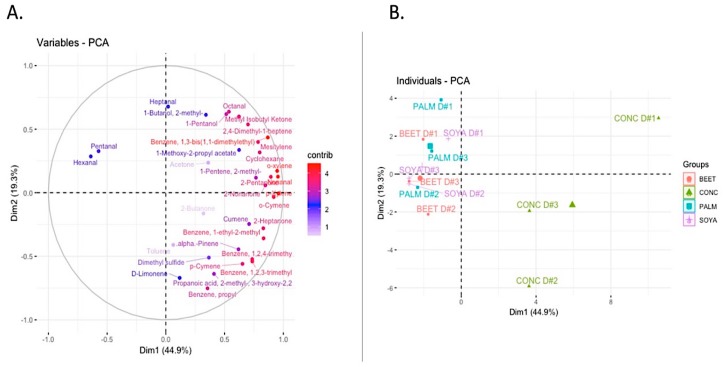
Principal component analysis of volatile compounds detected from milks from each of the diets, parlour concentrate (CONC), palm kernel expeller (PALM), soya hulls (SOYA), and molassed beet pulp (BEET).

Volatile analysis of milks using untargeted GC-MS SPME technique identified 34 compounds present in each of the samples (Table 9). Measurement of the volatiles in the headspace of products provides a representative view of the volatiles relative to the olfactory response [34]. There are a variety of factors that can impact the volatile profile of milk. A comprehensive review of the topic has highlighted a direct transfer of many volatiles from grazing or from conserved forage, some of which are related to plant (forage) metabolism or fermentation during conservation processes but many volatiles are also transferred indirectly post rumen metabolism [35]. In total, 33 compounds were detected in milks from each of the treatments from a variety of chemical groups including benzenes (10), ketones (6), sulfur compounds (1), alkenes (2), aldehydes (5), terpenes (4), and alcohols (2). Principal component analysis of the data revealed that the volatile profile of the CONC diet was distinctly different to the other supplementary feeding systems (Figure 2). A number of volatiles were identified to be responsible for this separation (Figure 2A). Dietary treatment was demonstrated to have a significant effect on the volatile profile of milks. Pentanal and hexanal are both aldehyde compounds typically derived from the degradation and oxidation of fatty acids. Pentanal was present at significantly (*P* < 0.05) higher concentrations in PALM than that of BEET and CONC, while levels of pentanal in SOYA were also significantly higher than that of CONC. Hexenal was significantly higher in BEET-, PALM-, and SOYA-derived milks than that of CONC. Hexanal mostly results from the degradation of oleic acid and linoleic acid, while pentenal can be derived from the oxidation of arachidonic and linoleic acid. Indeed, the levels of pentanal in milks were significantly and positively correlated with concentration of milk linoleic acid (*P* = 0.019, *r* = 0.661). The volatile p-xylene was present at significantly higher levels in CONC than that of BEET, PALM, and SOYA. The presence of p-xylene may be the result of carotenoid degradation, namely ß-carotene degradation [36], or possibly directly transferred from the feed [37].

### 3.4. Native Casein Micelle Size and Milk Gelation Properties

Feeding system was demonstrated to have a significant effect (*P* = 0.013) on the size of casein micelles and the gelation properties of the milks (Table 10). All z-average size distributions showed normal, mono-modal distributions. The CONC diet milks had the largest micelle size (167 nm), which was significantly greater than that of SOYA milk (153 nm) (*P* = 0.011), while the micelle size of PALM milks (163 nm) was also greater than that of SOYA (*P* = 0.058). Devold et al. [38] reported a similar trend of effect of feeding regimen on casein micelle size, whereby cows fed an ecologically rolled barley supplement produced significantly larger casein micelles than cows fed commercial supplementary concentrates. While genetic factors are typically reported to determine the size of casein micelles, micelle size is also affected by heat treatment, which causes pH-dependent interactions between β-lactoglobulin and κ-casein (on or off the casein micelle). Such factors impact on the volume fraction and packing arrangements of interacting casein micelles. The presence of minerals, especially calcium, also affect micellar interactions through neutralization of charge repulsion-induced stability. Neither of these influences were determined in the present study but should be considered in the future.

Alterations to casein micelle size and other characteristics of milk can be an important consideration as it has been previously demonstrated to play a significant role in the gelation properties of milks [39]. Glantz et al. [39] showed that milk gelation was affected by pH, protein profile, casein micelle size, and lactose. Gelation of milk forms the basis for the production of stabilized, high-value dairy products such as yoghurt, cheese, and their variants. There was a significant effect between feeding systems on the gelation properties of milks (*P* ≤ 0.01). The storage modulus of SOYA-derived milks was significantly higher than each of the other diets, storage modulus of SOYA > PALM > BEET > CONC, respectively. The CONC milk had the lowest storage modulus, significantly lower than that of PALM (*P* = 0.016) and SOYA (*P* ≤ 0.01). A similar trend was observed for loss modulus measurement whereby SOYA had significantly higher scores than BEET (*P* = 0.003) and CONC (*P* = 0.001), loss modulus of SOYA > PALM > BEET > CONC.

Gelation properties of milks can be influenced by a variety of factors including protein profile, milk composition, and stage of lactation. It seems that differences in the gelation properties observed in this study correlated with diet-induced changes to casein micelle size. Indeed, there was a significant negative correlation between casein micelle size and storage and loss modulus values (*P* = 0.009, *r* = −0.712 and *P* = 0.014, *r* = −0.686, respectively). It has been demonstrated, however, that combined changes in the casein micelle size and MFG size can influence curd firmness [40]. While the links between cow feeding system and casein micelle size requires further investigation, given these findings on impact of cow diet supplementation on milk gelation properties, dairy manufacturers should be aware of the types of dietary supplements used on farms and the potential implications for downstream product properties.

### 3.5. Colour Analysis

Diet did not have a significant effect on the L and a* colour scores on the milks, however there was a significant dietary effect on the b* values whereby CONC samples were significantly higher than that of BEET (*P* = 0.03). Increased b* values are indicative of a more yellow colour between the products. Typically, the yellowness of dairy products is attributed to the levels of β-carotene in the milks. β-carotene content of milk has been shown to vary with diet previously, and is typically higher in a pasture-derived diet, as processing of concentrates and ensiling of pasture depletes or destroys carotenoids [41], therefore resulting in a more white colour of products particularly in concentrated systems such as butter and cheese. Considering downstream final product quality, difference in colour of milks could be an important consideration, as increased yellow in products has been demonstrated to have a positive effect on consumer “liking of appearance” as demonstrated by O’Callaghan et al. [6] when examined by Irish dairy consumers.

## 4. Conclusions

Overall, this study has highlighted a variety of potential implications that choice of supplemented feed for a pasture-based cow diet can have on the composition, quality, and sensory aspects of milk. Supplemental feeding system was demonstrated to have a significant effect on a variety of fatty acids and subsequent indices, including total saturated and unsaturated fatty acids, *de novo*-produced FA, omega 3, and omega 6 FA. The volatile profile of milks was also affected by supplemental feed choice, whereby multivariate analysis demonstrated that the CONC diet was distinctly different to that of the PALM, SOYA, and BEET milks. Multivariate analysis highlighted the ability to distinguish milks between pasture-supplemented feeding systems by their fatty acid profile.

## Figures and Tables

**Table 1:** Concentrate feed allocation and forage allowance (grazed grass and grass silage) offered daily throughout the experimental period.

Feeding System	Parlour Concentrate (kg of DM/d)	Additional Supplement (kg of DM/d)	Grass Silage (kg of DM/d)	Grazed Grass (kg of DM/d)	Total DMI^1^ (kg of DM/d)
**Parlour Concentrate (CONC)**	2.00	0.00	9.00	5.00	16.00
**Palm Kernel Expeller (PALM)**	2.00	4.00	5.00	5.00	16.00
**Soya Hulls (SOYA)**	2.00	4.00	5.00	5.00	16.00
**Molasses Beet Pulp (BEET)**	2.00	4.00	5.00	5.00	16.00

DMI^1^ = Dry matter intake; DM: Dry matter.

**Table 2:** Chemical composition (g/kg of dry matter (DM); mean ± standard deviation (SD) and nutritional content of the grazed grass and grass silage collected weekly throughout the experimental period.

	Grazed Grass	Grass Silage
**Dry Matter**	233.2 ± 38.93	310 ± 41.45
**Organic Matter Digestibility**	799.7 ± 39.20	731.0 ± 11.13
**Crude Protein**	222.5 ± 25.59	139.0 ± 10.55
**ADF^1^**	276.9 ± 27.56	291.9 ± 21.40
**NDF^2^**	525.0 ± 33.93	450.0 ± 39.31
**Ash**	89.7 ± 22.75	104.0 ± 9.71

ADF^1^ = Acid detergent fibre; NDF^2^ = Neutral detergent fibre.

**Table 3:** Chemical composition (g/kg of DM; mean ± SD) of the supplemented feed collected weekly throughout the experimental period.

	CONC	PALM	SOYA	BEET
**Dry Matter**	869 ± 1.6	911 ± 22.7	869 ± 6.7	876 ± 2.4
**Organic Matter Digestibility**	-	657 ± 70.2	800 ± 58.1	871 ± 59.7
**Crude Protein**	163.7 ± 0.8	149.2 ± 9.2	93.8 ± 0.8	84.5 ± 0.8
**Crude Fibre**	51.0 ± 0.9	199.0 ± 32.3	360.0 ± 7.5	120.5 ± 5.8
**ADF^1^**	-	539 ± 34.1	446 ± 21.2	202 ± 17.3
**NDF^2^**	248 ± 40.8	810 ± 41.2	622 ± 33.1	401 ± 24.8
**Oil**	26.8 ± 0.8	71.7 ± 10.1	19.0 ± 2.3	8.7 ± 0.80
**Ash**	81.3 ± 0.8	37.7 ± 2.6	42.5 ± 0.8	99.8 ± 1.2
**Free Fatty Acid**	4.5 ± 0.50	23.0 ± 7.3	2.0 ± 0.01	2.0 ± 0.02

ADF^1^ = Acid detergent fibre; NDF^2^ = Neutral detergent fibre; CONC: Parlour Concentrate; PALM: Palm Kernel Expeller; SOYA: Soya Hulls; BEET: Molasses Beet Pulp.

**Table 4:** The effect of dietary treatment (parlour concentrate, palm kernel expeller, soya hulls, and molassed beet pulp) on milk production and milk composition, during the experimental period (23 July to 31 August).

	CONC	PALM	SOYA	BEET	SE	*P*-Value
**Daily Milk Yield (kg/cow/day)**	19.2 ^a^	19.8 ^ab^	21.4 ^c^	21.4 ^bc^	0.27	0.001
**Milk Fat (g/kg)**	43.3 ^a^	48.8 ^b^	45.4 ^a^	44.8 ^a^	1.01	0.01
**Milk Protein (g/kg)**	35.7	35.3	35.2	35.0	0.43	NS
**Milk Solids Yield (kg/cow/day)**	1.52 ^a^	1.64 ^bc^	1.72 ^b^	1.60 ^ac^	0.029	0.001

a,b and c denote significant difference between feeding systems.

**Table 5:** Feed supplements mean major fatty acid triglyceride content (% of total fatty acids (TFA)). Parlour concentrate (CONC), palm kernel expeller (PALM), soya hulls (SOYA), and molassed beet pulp (BEET).

Fatty Acid (% of TFA)	CONC	PALM	SOYA	BEET
**Caprylic Acid-C8:0**	<0.05	3.25	<0.05	<0.05
**Capric Acid-C10:0**	<0.05	3.31	<0.05	0.31
**Lauric Acid-C12:0**	0.16	47.24	0.29	2.36
**Myristric Acid-C14:0**	0.12	16.22	0.23	1.35
**Pentadecanoic Acid-C15:0**	0.06	<0.05	0.14	0.32
**Palmitic Acid-C16:0**	13.53	8.47	11.85	29.61
**Stearic Acid-C18:0**	2.25	2.55	4.48	2.13
**Oleic Acid-C18:1**	27.36	15.81	20.71	20.65
**Linoleic Acid-C18:2**	48.26	2.58	47.39	34.28
**Linolenic Acid-C18:3**	2.82	<0.05	9.44	4.72
**Stearidonic Acid-C18:4**	0.06	<0.05	0.1	0.27
**Arachidic Acid-C20:0**	0.44	0.14	0.54	0.28
**Gadoleic Acid-C20:1**	0.46	0.11	0.29	0.38
**Behenic Acid-C22:0**	0.26	<0.05	0.48	0.48
**Lignoceric Acid-C24:0**	0.17	0.08	0.23	0.75

**Table 6:** The effect of dietary treatment (parlour concentrate (CONC), palm kernel expeller (PALM), soya hulls (SOYA), and molassed beet pulp (BEET)) on bulk milk composition (% ± standard deviation).

	CONC	PALM	SOYA	BEET	*P*-Value
**Crude Protein (%)**	3.87 ± 0.20	3.83 ± 0.14	3.87 ± 0.10	3.79 ± 0.09	0.932
**Fat (%)**	4.6 ± 0.07	4.75 ± 0.13	4.47 ± 0.25	4.49 ± 0.15	0.352
**Lactose (%)**	4.62 ± 0.05	4.57 ± 0.06	4.66 ± 0.02	4.56 ± 0.04	0.186
**Total solids (%)**	13.88 ± 0.18	13.81 ± 0.21	13.7 ± 0.2	13.55 ± 0.13	0.356
**Whey protein (%)**	0.62 ± 0.01	0.61 ± 0.00	0.59 ± 0.01	0.57 ± 0.013	0.001
**Casein (%)**	3.08 ± 0.18	3.04 ± 0.15	3.11 ± 0.11	3.06 ± 0.11	0.963
**True Protein (%)**	3.7 ± 0.18	3.66 ± 0.15	3.7 ± 0.10	3.63 ± 0.10	0.939
**Total N (%)**	0.61 ± 0.03	0.6 ± 0.02	0.61 ± 0.02	0.59 ± 0.01	0.932
**Non Casein N (%)**	0.12 ± 0.00	0.12 ± 0.00	0.12 ± 0.00	0.11 ± 0.00	0.014
**Non Protein N (%)**	0.03 ± 0.00	0.03 ± 0.00	0.03 ± 0.00	0.03 ± 0.00	0.570

**Table 7:** Milk fatty acid composition (g/100g of FA) from cows feed pasture diets supplemented with CONC, PALM, SOYA, or BEET.

Fatty Acids	CONC	PALM	SOYA	BEET	*P*-Value
**Butyric acid C4:0**	4.06 ± 0.07	3.87 ± 0.05	4.15 ± 0.10	3.88 ± 0.03	0.01
**Caproic acid C6:0**	2.63 ± 0.02	2.49 ± 0.05	2.71 ± 0.01	2.63 ± 0.02	<0.001
**Octanoic acid C8:0**	1.52 ± 0.01	1.45 ± 0.02	1.56 ± 0.01	1.56 ± 0.02	<0.001
**Decanoic acid C10:0**	3.4 ± 0.04	3.26 ± 0.02	3.45 ± 0.02	3.61 ± 0.06	<0.001
**Undecanoic acid C11:0**	0.09 ± 0.01	0.1 ± 0.01	0.08 ± 0.01	0.09 ± 0.01	0.04
**Lauric Acid C12:0**	4.31 ± 0.18	7.33 ± 0.49	4.32 ± 0.14	4.46 ± 0.12	<0.001
**Tridecanoic acid C13:0**	0.11 ± 0.01	0.11 ± 0.01	0.1 ± 0.01	0.11 ± 0.01	0.19
**Myristic acid C14:0**	12.75 ± 0.11	14.22 ± 0.12	13.04 ± 0.12	13.09 ± 0.05	<0.001
**Myristoleic acid C14:1 (c9)**	1.24 ± 0.11	1.39 ± 0.03	1.21 ± 0.08	1.4 ± 0.07	0.07
**Pentadecanoic acid C15:0**	1.41 ± 0.10	1.28 ± 0.04	1.32 ± 0.01	1.39 ± 0.01	0.16
**Palmitic acid C16:0**	36.48 ± 1.37	34.93 ± 1.21	36.9 ± 0.48	38.94 ± 0.33	0.02
**Palmitoleic acid C16:1 (c9)**	1.74 ± 0.15	1.79 ± 0.09	1.79 ± 0.07	2.03 ± 0.10	0.10
**Heptadecanoic acid C17:0**	0.62 ± 0.01	0.48 ± 0.01	0.6 ± 0.01	0.58 ± 0.02	<0.001
**cis-10-Heptadecanoic acid C17:1 (c10)**	0.01 ± 0.01	0 ± 0.0	0.01 ± 0.0	0.01 ± 0.01	0.03
**Stearic acid C18:0**	9.22 ± 0.74	8.49 ± 0.34	8.91 ± 0.34	7.37 ± 0.35	0.02
**Elaidic acid C18:1 n9t**	0.28 ± 0.04	0.2 ± 0.05	0.31 ± 0.01	0.22 ± 0.01	0.04
**Oleic acid C18:1 n9c**	16.82 ± 0.6	15.91 ± 0.57	16.1 ± 0.19	15.25 ± 0.3	0.05
**trans-9,12-octadecadienoate C18:2 n6t**	0.12 ± 0.01	0.17 ± 0.01	0.12 ± 0.01	0.11 ± 0.01	<0.001
**Linoleic acid C18:2 n6c**	1.00 ± 0.03	0.82 ± 0.02	1.22 ± 0.16	1.14 ± 0.01	0.01
**Gamma Linolenic Acid C18:3 n6**	0.01 ± 0.01	0.02 ± 0.01	0.01 ± 0.01	0.02 ± 0.01	0.09
**Eicosanoic acid C20:0**	0.13 ± 0.01	0.11 ± 0.01	0.12 ± 0.01	0.11 ± 0.01	0.02
**α-Linolenic acid C18:3 n3**	0.66 ± 0.02	0.44 ± 0.03	0.64 ± 0.03	0.62 ± 0.04	<0.001
**CLA C18:2 c9t11**	0.96 ± 0.04	0.79 ± 0.05	0.92 ± 0.05	1.01 ± 0.03	0.01
**CLA C18:2 c10t12**	0.12 ± 0.01	0.1 ± 0.01	0.11 ± 0.01	0.11 ± 0.01	0.09
**cis-11-Eicosenoic acid C20:1**	0.02 ± 0.01	0.03 ± 0.01	0.03 ± 0.01	0.02 ± 0.01	0.42
**Behenic acid C22:0**	0.07 ± 0.01	0.06 ± 0.01	0.08 ± 0.01	0.07 ± 0.01	0.02
**Arachidonic acid C20:4**	0.05 ± 0.01	0.04 ± 0.01	0.05 ± 0.01	0.05 ± 0.01	0.01
**Docosadienoic Acid C22:2**	0.08 ± 0.01	0.07 ± 0.01	0.07 ± 0.01	0.07 ± 0.01	0.01
**Tetracosanoic acid C24:0**	0.04 ± 0.01	0.03 ± 0.01	0.04 ± 0.01	0.04 ± 0.01	<0.001
**cis-15-Tetracosenoic acid C24:1**	0.01 ± 0.01	0.01 ± 0.01	0.01 ± 0.01	0.01 ± 0.01	0.73
**cis-4,7,10,13,16,19-Docosahexaenoic acid C22:6**	0.02 ± 0.01	0.02 ± 0.01	0.03 ± 0.01	0.02 ± 0.01	0.64

CLA: conjugated linoleic acid.

**Table 8:** Indices of fatty acid profiles from milks derived from cows fed a pasture diet supplemented with CONC, PALM, SOYA, or BEET.

Fatty Acid Indices	CONC	PALM	SOYA	BEET	*P*-Value
**SFA**	76.85 ± 0.42	78.21 ± 0.5	77.36 ± 0.14	77.91 ± 0.4	0.03
**UFA**	23.15 ± 0.42	21.79 ± 0.5	22.64 ± 0.14	22.09 ± 0.4	0.03
**PUFA**	3.03 ± 0.01	2.47 ± 0.03	3.18 ± 0.15	3.15 ± 0.03	<0.001
**MUFA**	20.12 ± 0.43	19.33 ± 0.52	19.46 ± 0.06	18.94 ± 0.38	0.08
**PUFA/SFA**	0.04 ± 0.01	0.03 ± 0.01	0.04 ± 0.01	0.04 ± 0.01	<0.001
**MUFA/SFA**	0.26 ± 0.01	0.25 ± 0.01	0.25 ± 0	0.24 ± 0.01	0.07
**Hardness Index (C18:1n–9 cis/C16:)**	0.46 ± 0.03	0.46 ± 0.03	0.44 ± 0.01	0.39 ± 0.01	0.07
**Short Chain (C4–C14)**	30.11 ± 0.26	34.21 ± 0.49	30.6 ± 0.29	30.82 ± 0.08	<0.001
**Medium Chain (C15–C17)**	40.25 ± 1.58	38.49 ± 1.31	40.62 ± 0.54	42.95 ± 0.35	0.02
**Long Chain (C18–C24)**	29.64 ± 1.39	27.3 ± 0.85	28.77 ± 0.62	26.23 ± 0.34	0.02
**Ω-3**	0.69 ± 0.03	0.46 ± 0.03	0.66 ± 0.03	0.64 ± 0.04	0.00
**Ω-6**	1.14 ± 0.03	0.94 ± 0.03	1.36 ± 0.16	1.27 ± 0.02	0.01
**Ω-9**	17.14 ± 0.65	16.14 ± 0.56	16.45 ± 0.19	15.51 ± 0.29	0.04
**Ω-3/Ω-6**	0.6 ± 0.03	0.49 ± 0.04	0.5 ± 0.07	0.5 ± 0.03	0.14
**AI**	4.18 ± 0.13	4.79 ± 0.15	4.35 ± 0.07	4.6 ± 0.10	<0.001
**TI**	4.4 ± 0.10	4.81 ± 0.19	4.56 ± 0.04	4.75 ± 0.17	0.07
**C14:/C14:0**	0.1 ± 0.01	0.1 ± 0.01	0.09 ± 0.01	0.11 ± 0.01	0.18
**C16:1/C16:0**	0.05 ± 0.01	0.05 ± 0.01	0.05 ± 0.01	0.05 ± 0.01	0.16
**Σ18:1/C18:0**	1.86 ± 0.08	1.9 ± 0.04	1.84 ± 0.05	2.11 ± 0.12	0.04
**LA/ALA**	1.51 ± 0.07	1.89 ± 0.18	1.93 ± 0.34	1.85 ± 0.13	0.24
***De novo* (C4–C14)**	28.87 ± 0.29	32.82 ± 0.52	29.40 ± 0.28	29.42 ± 0.06	<0.001

SFA: saturated fatty acids; UFA: unsaturated fatty acids; PUFA: poly unsaturated fatty acids; MUFA: mono-unsaturated fatty acids; TI: thrombogenic; AI: atherogenic; LA: linoleic acid.

**Table 9:** Volatile profile of raw milks derived from pasture feeding systems supplemented with CONC, BEET, SOYA, or PALM.

Compound	CAS Number	LRI	Ref LRI	CONC	BEET	SOYA	PALM	SEM
Acetone	67-64-1	527	536	2.27 × 10^8^	1.81 × 10^8^	1.74 × 10^8^	2.47 × 10^8^	1.19 × 10^7^
Dimethyl sulfide	75-18-3	531	535	7.69 × 10^5^	3.83 × 10^5^	5.65 × 10^5^	5.41 × 10^5^	6.34 × 10^4^
1-Pentene, 2-methyl-	763-29-1	588	594	1.36 × 10^6^	9.67 × 10^5^	9.12 × 10^5^	8.70 × 10^5^	7.96 × 10^4^
2-Butanone	78-93-3	635	635	4.00 × 10^6^	2.59 × 10^6^	3.27 × 10^6^	4.13 × 10^6^	2.37 × 10^5^
Cyclohexane	110-82-7	664	670	3.12 × 10^6^	1.95 × 10^6^	2.14 × 10^6^	2.08 × 10^6^	2.08 × 10^5^
2-Pentanone	107-87-9	726	726	3.22 × 10^6^	1.97 × 10^6^	1.75 × 10^6^	1.92 × 10^6^	2.51 × 10^5^
Pentanal	110-62-3	731	733	5.34 × 10^6^	8.07 × 10^6^	1.10 × 10^7^	1.45 × 10^7^	1.10 × 10^6^
Methyl Isobutyl Ketone	108-10-1	777	784	9.19 × 10^6^	7.94 × 10^6^	6.61 × 10^6^	7.78 × 10^6^	6.65 × 10^5^
1-Butanol, 2-methyl-	137-32-6	781	789	3.83 × 10^6^	2.48 × 10^6^	3.63 × 10^6^	4.14 × 10^6^	3.01 × 10^5^
Toluene	108-88-3	788	796	6.81 × 10^7^	4.48 × 10^7^	7.78 × 10^7^	3.58 × 10^7^	1.08 × 10^7^
1-Pentanol	71-41-0	815	813	1.32 × 10^6^	1.00 × 10^6^	1.20 × 10^6^	1.23 × 10^6^	1.32 × 10^5^
Hexanal	66-25-1	835	837	1.02 × 10^7^	1.95 × 10^7^	2.53 × 10^7^	1.78 × 10^7^	1.78 × 10^6^
2,4-Dimethyl-1-heptene	19549-87-2	846	850	2.85 × 10^6^	1.74 × 10^6^	2.26 × 10^6^	2.42 × 10^6^	1.99 × 10^5^
p-Xylene	106-42-3	893	896	5.92 × 10^6^	2.92 × 10^6^	2.94 × 10^6^	3.10 × 10^6^	4.40 × 10^5^
1-Methoxy-2-propyl acetate	108-65-6	899	898	1.43 × 10^6^	1.09 × 10^6^	1.19 × 10^6^	9.72 × 10^5^	9.28 × 10^4^
o-xylene	95-47-6	921	925	3.07 × 10^6^	1.76 × 10^6^	1.83 × 10^6^	1.81 × 10^6^	2.10 × 10^5^
2-Heptanone	110-43-0	930	932	6.32 × 10^6^	3.12 × 10^6^	3.50 × 10^6^	3.70 × 10^6^	4.62 × 10^5^
Heptanal	111-71-7	938	940	1.80 × 10^6^	2.11 × 10^6^	2.16 × 10^6^	2.33 × 10^6^	1.39 × 10^5^
α-Pinene	80-56-8	948	952	3.98 × 10^6^	2.37 × 10^6^	2.20 × 10^6^	2.17 × 10^6^	3.71 × 10^5^
Benzene, propyl	103-65-1	979	980	6.93 × 10^5^	5.39 × 10^5^	5.17 × 10^5^	4.83 × 10^5^	4.58 × 10^4^
Cumene	98-82-8	987	991	4.93 × 10^5^	3.50 × 10^5^	3.66 × 10^5^	3.90 × 10^5^	2.19 × 10^4^
Benzene, 1,2,3-trimethyl	526-73-8	990	994	4.51 × 10^5^	2.90 × 10^5^	3.04 × 10^5^	2.93 × 10^5^	2.41 × 10^4^
Benzene, 1,2,4-trimethyl	95-63-6	991	994	4.50 × 10^5^	2.90 × 10^5^	3.04 × 10^5^	2.93 × 10^5^	2.40 × 10^4^
Benzene, 1-ethyl-2-methyl	611-14-3	1022	1009	4.55 × 10^5^	3.28 × 10^5^	3.10 × 10^5^	3.28 × 10^5^	2.11 × 10^4^
Mesitylene	108-67-8	1023	1030	3.93 × 10^6^	3.07 × 10^6^	3.23 × 10^6^	3.09 × 10^6^	2.15 × 10^5^
Octanal	124-13-0	1041	1053	1.86 × 10^6^	1.69 × 10^6^	1.44 × 10^6^	1.79 × 10^6^	2.05 × 10^5^
D-Limonene	5989-27-5	1047	1056	7.09 × 10^5^	4.84 × 10^5^	5.49 × 10^5^	4.64 × 10^5^	6.09 × 10^4^
o-Cymene	527-84-4	1050	1056	2.08 × 10^6^	8.36 × 10^5^	7.45 × 10^5^	8.55 × 10^5^	1.92 × 10^5^
p-Cymene	99-87-6	1080	1084	1.98 × 10^5^	1.64 × 10^5^	1.60 × 10^5^	1.47 × 10^5^	7.95 × 10^3^
2-Nonanone	821-55-6	1134	1142	1.87 × 10^6^	1.06 × 10^6^	1.07 × 10^6^	9.58 × 10^5^	1.57 × 10^5^
Nonanal	124-19-6	1144	1151	2.83 × 10^6^	1.96 × 10^6^	1.97 × 10^6^	2.07 × 10^6^	1.38 × 10^5^
Benzene, 1,3-bis(1,1-dimethylethyl)	1014-60-4	1279	1288	1.48 × 10^7^	6.70 × 10^6^	8.81 × 10^6^	9.41 × 10^6^	1.36 × 10^6^
Propanoic acid, 2-methyl-, 3-hydroxy-2,2	77-68-9	1454	1465	8.29 × 10^5^	7.28 × 10^5^	4.52 × 10^5^	3.59 × 10^5^	6.64 × 10^4^

LRI: Linear retention indice; SEM: Standard error of mean.

**Table 10:** Summary of effect of diet on the casein micelle size and gel strength of milks derived from BEET, CONC, PALM, and SOYA.

Diet	BEET	CONC	PALM	SOYA	*P*-Value
Average Z-size (nm)	158.79 ± 2.25	167.27 ± 5.05	163.41 ± 2.71	153.34 ± 2.06	0.013
Storage modulus (Pa)	43.52 ± 3.01	26.63 ± 7.54	60.61 ± 13.77	91.9 ± 5.54	<0.001
Loss modulus (Pa)	17.69 ± 1.47	11.58 ± 3.02	25.18 ± 5.42	36.56 ± 3.22	0.001

CONC: Parlour Concentrate; PALM: Palm Kernel Expeller; SOYA: Soya Hulls; BEET: Molasses Beet Pulp.

## Supplementary Materials

The following are available online at https://www.mdpi.com/2304-8158/8/4/137/s1, Figure S1: Top 10 fatty acids significantly contributing to observed PCA separation.
