# Supplementary material for: Influence of Supplemental Feed Choice for Pasture-Based Cows on the Fatty Acid and Volatile Profile of Milk
Source: Foods. 2019 Apr 22;8(4):137. doi: 10.3390/foods8040137 (PMC6518226; doi:10.3390/foods8040137)

Supplementary Figure 1: Top 10 fatty acids significantly contributing to observed PCA separation

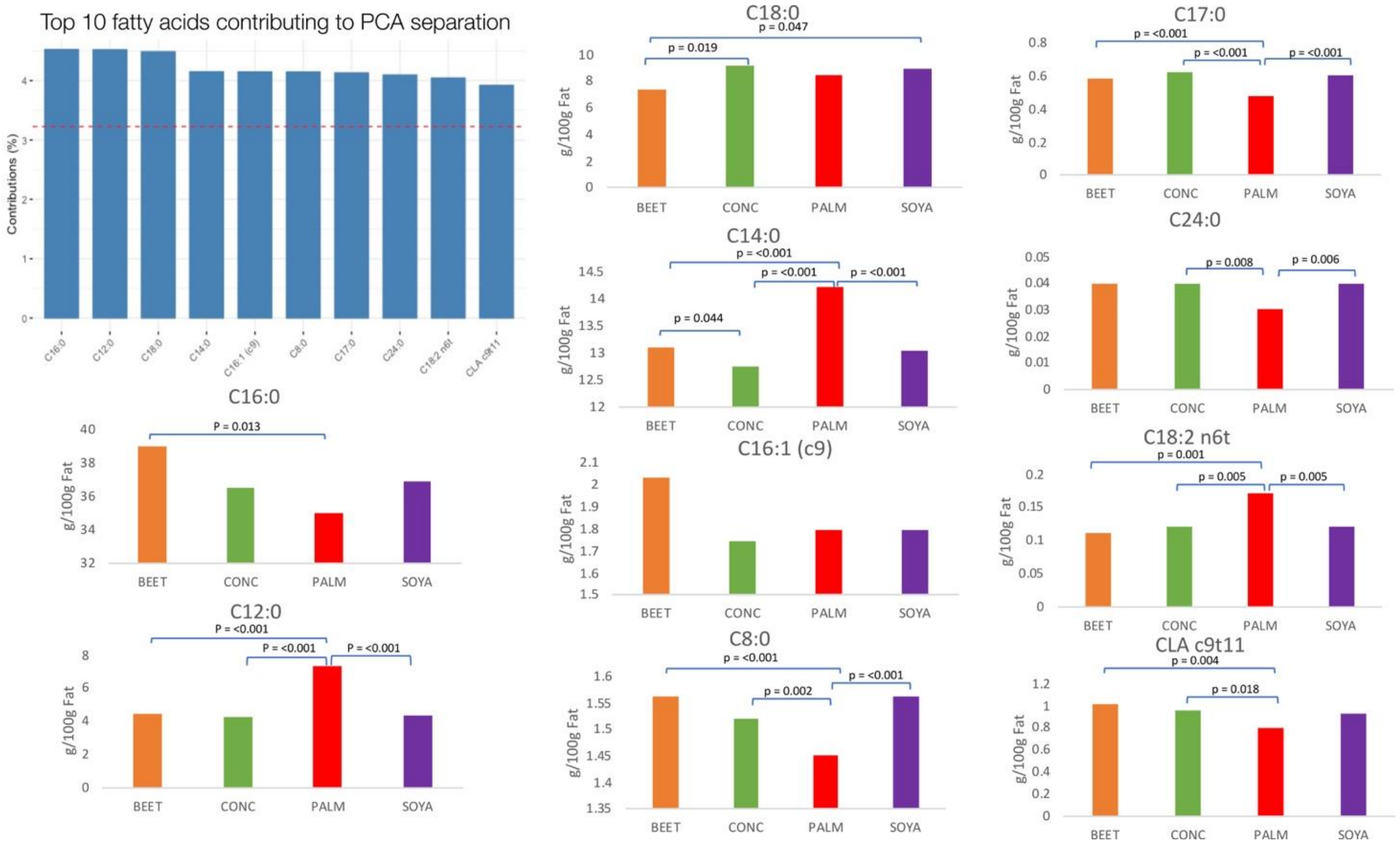

Supplement: Supplementary file 1 [file foods-08-00137-s001.pdf]
